# Preface

**Published:** 1824-06-01

**Authors:** 


### PREFACE.

AT the commencement of a New Series, some prefatory observations will naturally be expected. The encouragement of the Public might seem to demand our sincere thanks—but we doubt whether the Public care much about them. They look for *doings* rather than *sayings*. We apprehend that, if we came before them quarterly with a laudatory preface and a lean table of contents, we should soon preach to the "viewless winds." Under this impression, we have thought it proper to express our gratitude in substantial facts, rather than in specious promises. We chuse to address the SENSE, rather than the PASSIONS of our readers—their judgment rather than their imaginations—their interest rather than their amusement. Whether this plan, which we have uniformly endeavoured to pursue since the commencement of our labours, has been hitherto, or is likely to be in future, crowned with success, we have no inclination, at present, to enquire. The facts of the case are best known to those most interested in the question: and we leave it to be settled by our friends and our enemies in whatever way it may be most agreeable to them.

If it be asked, why we have begun a New Series? the answer is, that the back numbers are out of print, and few people like to begin with a broken set of a periodical journal. But why not reprint them? Because such a re-publication would require much time, much trouble, and great expence. And, after all, many, who would wish to commence with a New Series, would not choose to go to the expence of four years' back numbers.* By the present plan, we occasion no inconvenience to the holders of the former series, since the numbers and volumes are still continued; while we afford a great facility to those who subscribe from the present time.

It has been represented to us, that there is great danger in closing a series. Old friends may wish to change—and at such a period they have a good opportunity. Be it so. God forbid that we should throw any obstacle in the way of those who wish to quit our company. They are now released from all restraints or obligations, and may depart in peace.

But, as we run the risk of closing one series, we may fairly claim the advantage of opening another. Many hundred young practitioners now annually start in the career of practice. The present may be characterized as the reading age. No practitioner henceforth can be without a periodical publication in medical science, and we stand before the Public among the candidates for a share of their patronage.

The scope and tendency of this Review are now well known. The plan was, at first, considered somewhat utopian. Time has proved that it is perfectly practicable. While unencumbered with original communications, our Periscope enables us to range through the whole galaxy, and cull out the choice morsels. But it will be said, we have them at second hand. So much the better. If these articles will not bear keeping for three or even six months, we had rather not ship them among our cargo. There is, in fact, great variety in these commodities. Some keep a week, and no more. The moment they see the light they perish. Others keep a month, and then lan-

* Those who do, will, we hope, soon be accommodated by sets of the work, (now reprinting in America) and ordered from thence.

guish. If they stand three months, they are worth trial, and we import the best samples from abroad to mix with the best specimens which we can procure at home.

So much for our Periscope. Most of the above observations will apply, also, to the main department of our work, the *Review of Books*. We venture to affirm, that we have carried on this department in a manner somewhat different from any that has ever been adopted before, by periodical writers. We have aimed at the very difficult task of exhibiting a comprehensive and connected view, combined with a minute and particular detail of the works which we have analyzed. This is a task which it is not so easy to accomplish as some people may imagine. We could not, for some time, effect it. No inconsiderable experience is necessary for its completion. Symptoms have been lately evinced which would lead us to suspect that a publication of this kind was not entirely unwelcome to the profession. Our brethren, scattered abroad in the Colonies, or distracted with the toils of private practice at home, cannot procure, and would not peruse (if procured) one tenth of the effusions of the press in their original forms. These effusions, foreign and domestic, generally require the literary filter, or alembic—and we are often astonished at the quantum of residue, or caput mortuum, which remains after the process is finished!

We have said that this is a *reading* age. It is preeminently a *writing* one also. It would almost seem that the chimerical project of equalizing ranks, rights, and riches, had now changed to the equally chimerical project of placing all classes of society on a level, in respect to knowledge. Thus we see some engines at work to debase the faculty in the eyes of the populace, while others are endeavouring to elevate the populace, (in their own eyes at least) to rank with the faculty in medical lore! What may be the various ultimate consequences of this attempt at EQUALITY in medical matters, we do not pretend to divine:—

But we apprehend, that it will be attended with one GOOD EFFECT at least—that of forcing all classes of medical society to an increased cultivation of the science they profess. Thus, while blunders may probably become more numerous among the people themselves, it is likely that *error will diminish among their medical attendants.*

In promotion of this last object, we have laboured with unfeigned zeal for many years—and we trust it will not be deemed presumption in us to express a hope, that our labours have not been entirely useless to our brethren and to society at large. The task of conducting a Medical REVIEW is at once delicate, dangerous, and difficult; but we have the gratification of knowing that our conduct has not yet been impeached in a single instance, (and we appeal for the truth of this to the profession at large) except by anonymous revilers, whose envious and unprincipled effusions we know full well (and so do they) to be the best species of applause. *These* we shall never, under any circumstance whatever, condescend to notice, content with the suffrages of the good from abroad—and with the "MENS CONSCIA RECTI" at home.

---

*Nota.* The first volume of Dr. Bostock's valuable Elementary System of Physiology came, after the Bibliogrrphical Record was closed. It is to be particularly remembered, that all Notices, Intelligence, Books for Review, &c. will be too late for acknowledgment after the 16th of the month preceding publication.

